# Antibodies inside of a cell can change its outside: Can intrabodies provide a new therapeutic paradigm?

**DOI:** 10.1016/j.csbj.2016.07.003

**Published:** 2016-07-31

**Authors:** Andrea L.J. Marschall, Stefan Dübel

**Affiliations:** a Department of Systems Immunology and Braunschweig Integrated Centre of Systems Biology, Helmholtz Centre for Infection Research, Braunschweig, Germany; b Institute of Biochemistry, Biotechnology and Bioinformatics, Technische Universität Braunschweig, Spielmannstr.7, 38106 Braunschweig, Germany

## Abstract

Challenges posed by complex diseases such as cancer, chronic viral infections, neurodegenerative disorders and many others have forced researchers to think beyond classic small molecule drugs, exploring new therapeutic strategies such as therapy with RNAi, CRISPR/Cas9 or antibody therapies as single or as combination therapies with existing drugs. While classic antibody therapies based on parenteral application can only reach extracellular targets, intracellular application of antibodies could provide specific advantages but is so far little recognized in translational research. Intrabodies allow high specificity and targeting of splice variants or post translational modifications. At the same time off target effects can be minimized by thorough biochemical characterization. Knockdown of cellular proteins by intrabodies has been reported for a significant number of disease-relevant targets, including ErbB-2, EGFR, VEGFR-2, Metalloproteinase MMP2 and MMP9, β-amyloid protein, α-synuclein, HIV gp120, HCV core and many others. This review outlines the recent advances in ER intrabody technology and their potential use in therapy.

## 1 Utilizing the specificity of antibodies inside of living cells

Intrabodies are antibodies expressed intracellularly to block cellular functions. In contrast to the naturally expressed antibodies which are secreted and directed towards extracellular targets, intracellularly expressed antibodies, are directed towards targets inside the cell. This allows utilizing the very high specificity of antibody/antigen binding for the functional analysis of proteins in living cells or even living organisms.

The use of antibodies in living cells started in the 1980s when they were found to be sufficiently stable after microinjection into the cytoplasm, and they were shown to be able to interfere with the function of their intracellular antigen. For instance, intermediate filaments were found to collapse after blocking their assembly with microinjected antibodies [8]. However, microinjection is laborious and allows only small cell numbers to be manipulated, which limited a widespread application of the technique. Hence, a number of approaches using reagents or peptides for protein delivery have been tried to introduce antibodies into living cells [33]. While the so called “cell penetrating peptides” (CPPs) gained considerable attention at early times after their discovery, their initially proposed mechanism of uptake and the general efficacy as transduction modules for macromolecules has meanwhile been questioned [33], [35], [39]. In contrast to initial assumptions, CPPs are now believed to be internalized by endocytosis if linked to macromolecules and the majority remains in endosomes, which may result in very low efficiency of cytosolic delivery [18], [33], [39]. Because inhibition of antigen function by the binding of antibodies to their antigen usually requires an at least 1:1 M ratio of the latter, this low efficiency of cytosolic delivery can substantially limit its applications for functional interference. Protein transfection (profection), which is based on reagents that are believed to possess properties which can enhance or trigger endosomal release [5], therefore has been suggested as a promising alternative [52]. However, similar to the initial difficulties to detect the true cytosolic release of cargo-molecules in research on CPPs, the efficiency of profection has recently been found to be largely overestimated too, due to the common usage of artifact-prone detection methods [35].

Despite the numerous attempts to deliver antibodies to the cytosol by using peptides or by means of profection, delivery into larger cell populations of amounts of antibodies comparable to the early microinjection experiments of the 1980s was only recently achieved by electroporation [15], [20], [35] and demonstrated that scFv-Fc antibodies (which are similar to the microinjected whole IgG but rely on the structural integrity of a scFv moiety) are functional for at least 96 h after electroporation into cells [35].

**Fig. 1:**
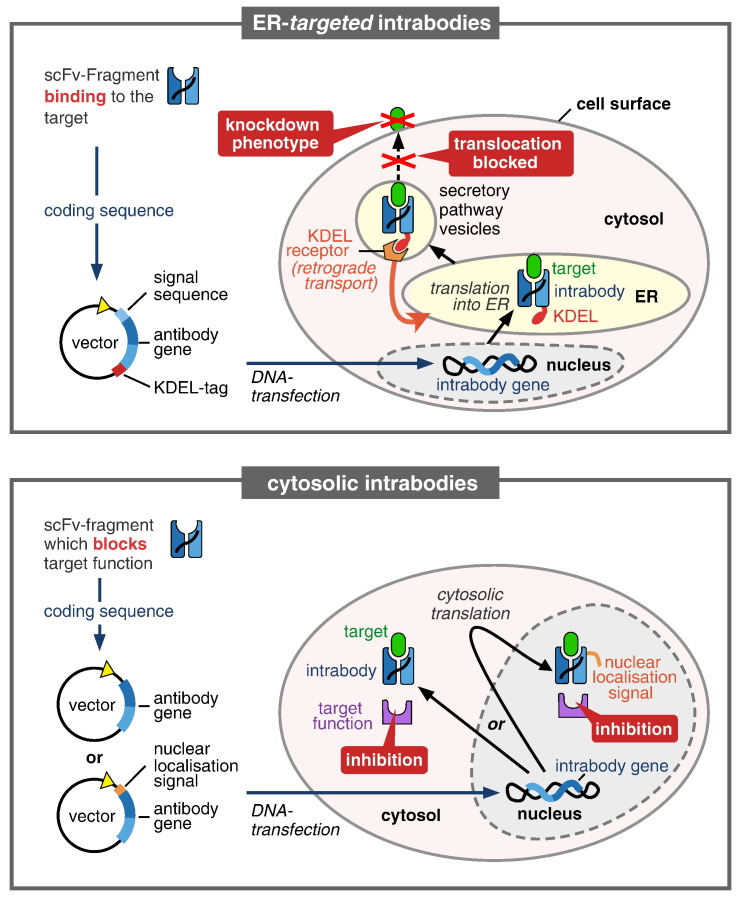
Properties and modes of action of intrabodies targeting antigens in the ER (top) or in the cytoplasm (bottom). ER targeted intrabodies require a signal sequence guiding them to be produced into the ER. There they cause a functional knockdown by binding to their target protein (antigen) inside of the ER and retaining it there, thus preventing it from reaching the cell surface or from being secreted. In order to achieve ER retention of a target protein, the intrabody itself needs to be retained in the ER, which is achieved by adding the ER retention signal peptide “KDEL”. Specific binding of an ER intrabody to its target is usually sufficient to provide the knockdown, while cytosolic intrabodies are usually required to additionally be inhibitory or blocking. Consequently, cytosolic intrabodies usually need to target a particular epitope. A second difference to ER intrabodies is that cytosolic intrabodies are translated in the cytosol, where the protein folding conditions for antibodies are less favorable than in the ER. Cytosolic intrabodies may also be targeted to the nucleus if provided with a nuclear localization sequence.

The limitations of protein delivery motivated attempts to express the genes of antibodies in cells early on [49]. As intrabody approaches work well with just the antigen binding fragments of an IgG, typically single chain Fv fragments (Fig. 1) or even single domain antibodies/nanobodies, they do not require assembly from two protein chains like the original antibody, eliminating the need for bicistronic vectors and the associated problems to achieve the correct heavy chain/light chain ratio upon expression. However, cytosolic expression of intrabodies did not always result in functional antibodies as many antibodies tend to misfold in the cytosol. This can be attributed to the reducing milieu preventing formation of disulfide bonds [6], [44] and lack of endoplasmic reticulum (ER) chaperones. In contrast, because antibodies are naturally secreted, their folding is optimal in the ER, which prompted the introduction of a different type of intrabodies: ER retained intrabodies. Functional knockdowns of membrane or secreted proteins can be achieved by means of ER retained intrabodies by providing a target specific antibody together with an ER retention signal, the amino acid sequence “KDEL” [29]. In this way, the intrabody is kept within the ER together with its target antigen. Trapping individual membrane proteins or secreted factors in the ER prevents these proteins from carrying out their normal function because they cannot reach their site of action anymore [32].

## 2 Different types of intrabodies

According to the different intracellular compartment for expression, intrabodies can be classified into two basic categories based on the requirements for their molecular structure: those that have to fold under reducing conditions and those that can form disulfide bonds in the environment in which they are expressed. While antibodies that are translated in the cytosol have to fold under reducing conditions, antibodies can form disulfide bonds in the ER and also in mitochondria [6]. Cytosolic intrabodies can be employed to target the cytosolic proteome as well as the nuclear proteome if a nuclear localization sequence is fused to the intrabody. ER intrabodies, in contrast, can only target secreted proteins. However, because many cellular processes are controlled by signaling via membrane receptors or secreted factors, being able to target the whole membranome and secretome provides the key to many crucial cellular processes.

The generation of the different types of intrabodies also requires very different amounts of effort. Although aggregating intrabodies have also been reported to cause a phenotype [12], cytosolic intrabodies, ideally i. need to fold correctly in the cytosol in order to be functional and ii. need to bind to a selected epitope in a way allowing neutralization of the target function. Because the majority of antibodies do not fold correctly in the cytosol, several strategies have been proposed to select for the rare antibodies that are stable and functional in the cytosol (for review see [32]). These strategies include the use of scaffolds that are known to be particularly suitable for folding correctly in the cytosol and onto which the antigen binding features of other antibodies are grafted [16]. Single domain antibodies like camelid nanobodies have also been employed particularly in the cytosol [25]. Fusion of intrabodies to other proteins with the aim to increase their solubility has also been suggested as a potential means for enhancing the cytosolic solubility of intrabodies [41]. However, because solubility alone is no guarantee for functionality and may even lower functionality in spite of higher solubility (such as lower fluorescence of a soluble GFP fusion to maltose binding protein (MBP) compared to unfused GFP in *E. coli*[22]), it is not clear whether this approach allows converting every non-functional intrabody into a functional one. In order to particularly screen for antibodies with properties that allow correct folding in the cytosol, *e.g.* antibodies that are stable even in the absence of disulfide bonds, various approaches relying on two hybrid-like and other in-cell interaction analysis techniques or quality control screenings have been developed (for review see [32]). Some of these screening technologies allow selection of intrabodies that are stable in the cytosol in an antigen-specific way [28], [48], [50], [51], [54], while other screening technologies allow to more generally screen for antibodies that are able to fold correctly in the cytosol independently of their antigen-specificity [3], [19].

Particularly strategies that aim at selecting for desired properties, such as selection for target binding in the cytosol or for correct folding are promising but quite laborious, besides reduced library complexities due to lower transfection efficiencies in yeast or mammalian cells as well as the generally reduced variety of cytosolically stable antibodies. An additional effort is necessary to select for antibodies with neutralizing properties, which are required for blocking functions. In contrast, the process for the generation of ER intrabodies is much more simple and straightforward. Because ER intrabodies are expressed in their natural compartment, there are no special requirements in respect of folding. Additionally, there is no need for ER intrabodies to be neutralizing, because binding to the target protein is sufficient to trap the target protein in the ER and thereby cause a functional knockdown by keeping it from reaching its site of action. While larger antibody formats such as full IgGs have been used in approaches to deliver antibodies as proteins to the cytosol, the smaller single chain Fragment variable (scFv), in which the antigen binding domains of heavy and light chain are connected by a peptide linker, has been more commonly applied for intracellular antibodies [32], [33]. Further, even single domain antibody fragments, such as camelid nanobodies, have been successfully employed as cytosolic intrabodies already (for review see [25]).

## 3 Intrabodies for therapy?

While obtaining the genes of monoclonal antibodies previously relied on the much more time consuming and labor intensive hybridoma technology, phage display now allows a much faster generation of antibodies [14], [36]. Especially the huge and still growing resource of well characterized antibodies from consortia such as “Affinomics” (http://cordis.europa.eu/result/rcn/90758_en.html), for which genes are already available, are ready to use for the knockdown of target proteins. Further, the easy availability of human antibodies [27] allows to mitigate potential immunogenicity issues of therapeutic intrabodies right from the start. And the potential benefits of intrabodies are quite attractive. While classic small molecule drugs are not available for all target proteins, antibodies have the potential for a much wider therapeutic application range because they can in principle be generated against all proteins of the proteome [14], [36]. Due to their large binding site, they can typically be much more specific than small molecules. Further, because they act at the protein level, they can target splice variants of target proteins, or single posttranslational modifications [7] which may allow therapeutic approaches not yet possible with other approaches. In contrast to classic antibody therapies based on the parenteral application of antibodies, therapy with intrabodies might allow further reduction of off target effects when put under control of tissue specific promotors. Furthermore, cytosolic intrabodies can reach intracellular proteins, which are not accessible to parenterally applied antibodies. By targeting one particular epitope of a protein that has several functions, it may also be possible to inhibit just one selected function of that target protein. In the case of cytosolic intrabodies, epitopes with different functions might be targeted, while in the case of ER intrabodies different epitopes can be targeted in order to knock down all splice variants of a protein or to knock down specifically one individual splice variant of a protein. This may allow therapy even where ubiquitous inhibition or knockout is not possible. Inhibition of one of the several functions of a target protein can furthermore be achieved by the ability of intrabodies fused to a signal sequence to relocate proteins, as has been demonstrated by the knockdown of Sec61, which was relocated intracellularly by an ER-intrabody to inhibit its function in the endosomes without disrupting its vital function for protein biosynthesis in the ER [53]. Another study reported the re-localization of a target protein to prevent its function in the nucleus [12]. A similar approach allowed to block the pathogenic polymerization of Z α1-antitrypsin while maintaining its antiproteinase activity without which patients would develop a lung disease [37].

Therapeutic approaches with cell type specificity or therapies that allow selective control of one of several functions of a target protein could be highly beneficial for making therapy more specific. Because intrabodies have the potential to provide such highly specific therapeutic approaches, *e.g.* by relocating a target protein or by targeting splice variants with intrabodies, they may increase safety by eliminating unwanted side effects that cannot be avoided by other methods. As antibodies are naturally produced in high amounts during an infection, the expression of ER intrabodies has so far been found to cause no significant ER stress in spite of substantial overexpression [37], [55]. This suggests that side effects originating from the expression of the foreign protein in a patient may also be negligible.

## 4 Therapeutic intrabodies need gene therapy

A critical factor for the therapeutic application of intrabodies, similar to the use of RNAi or CRISPR/Cas9 (for a review of the CRISPR/Cas9 system see [17], is the successful and safe introduction of DNA into cells in vivo [30]. Although clinical trials have been attempted for both intrabodies and RNAi, the majority of attempts has clearly been made to therapeutically harness RNAi [4]. This is most likely due to the so far much more laborious process (hybridoma technology) required to generate monoclonal antibodies, when compared to the relatively fast generation of RNAi sequences. However, with the many antibody sequences that are now available for instant use [14], [36], the challenges for therapeutic use of RNAi, CRISPR/Cas9 or intrabodies have become similar and mainly consist of the challenges generally associated with gene therapy (for a review on gene therapy see *e.g.*[26]). Because intrabodies act at the protein level, however, their therapeutic use allows novel therapeutic paradigms that cannot be achieved by RNA- or DNA- based approaches. And with approved gene therapy drugs on the market, there are robust and clinically proven delivery systems readily available which could be easily adapted to introduce intrabody genes into patient's cells.

*In vitro*, there are already many examples for successful intrabody mediated knockdowns, both achieved with cytosolic intrabodies and ER intrabodies. Targets that have been knocked down by intrabodies include oncogenic targets, proteins related to immune function, neuronal targets including those involved in neurodegenerative disorders and targets involved in chronic viral infections, showing the broad range of potential application of future therapeutic intrabodies [31]. A comprehensive overview is given in Ref. [32]. *In vivo*, successful intrabody mediated knockdowns have been demonstrated in a transgenic mouse in which the function of VCAM-1 was knocked down by ubiquitous expression of an ER intrabody as a transgene [34]. Further intrabody mediated knockdowns *in vivo* have been demonstrated via the therapeutically relevant delivery of genes by viral delivery vectors [1], [2], [24], [38], [45], [47].

Therapeutic strategies against chronic viral infections include intrabodies targeted directly against viral proteins or intrabodies that target host proteins [31]. An intrabody targeting the host protein CCR5 that is involved in viral entry of HIV into host cells, has for instance been found to protect cells from infection [43], [47]. An intrabody against the viral protein HIV Tat led to increased survival of CD4(+) T cells in rhesus macaques and in one animal even to a reduced viral load [9]. Furthermore, a very recent study reported on the reduction of infectious hepatitis C (HCV) viral titers in cell culture upon expression of an intrabody that probably interferes with viral assembly [46].

Neurodegenerative diseases have also been successfully targeted by intrabodies *in vitro* and *in vivo*[11]. For example an ER intrabody allowed interference with the maturation and glycosylation of prion protein [13], an intrabody inhibited the formation of high molecular weight species of alpha-Synuclein associated with Parkinson's disease [56] and an intrabody allowed interference with the aggregation and toxicity of a target associated with the killing of motorneurons in Amyotrophic Lateral Sclerosis, as demonstrated *in vitro*[21]. In a mouse model for Huntington's disease, viral delivery of an intrabody delayed the formation of aggregates and reduced pathology [42]. In a mouse model for Alzheimer's disease (AD), delivery of an intrabody gene by an adeno-associated vector reduced AD pathology on the molecular level as well as in the form of improved cognitive function [40].

In another recent study, retroviral delivery of an intrabody gene was reported to result in 60% of tumor-free mice compared to controls in a mouse model for tumors that are caused by human papilloma virus (HPV), demonstrating the potential of intrabodies for cancer therapy [2]. In conclusion, intrabodies will allow exploring completely new therapeutic strategies based on highly specific interactions at the protein level. These therapies will require somatic gene therapy, which is a well-established clinical practice now since the first approval of AAV based gene therapy in 2012 [23], not anymore representing a major obstacle. The AAV vectors have even been engineered already for tissue specific targeting [10], offering exciting opportunities to combine two independent disease related specificities from the outside and the inside to minimize off-target effects and increase safety.
